# Meta-analysis and systematic review of gout prevalence in the heart/lung transplantation population

**DOI:** 10.3389/frtra.2024.1356058

**Published:** 2024-05-20

**Authors:** Benedict Chui, Richard Day, Eshwar Umashankar, Christina Abdel Shaheed, Anne Keogh, Laila Girgis, Ross Penglase

**Affiliations:** ^1^ Faculty of Medicine and Health, St Vincent’s Clinical School, University of New South Wales, Sydney, NSW, Australia; ^2^ Department of Clinical Pharmacology, St Vincent’s Hospital, Sydney, NSW, Australia; ^3^ Sydney School of Public Health, Faculty of Medicine and Health, The University of Sydney, Sydney, NSW, Australia; ^4^ Institute for Musculoskeletal Health, University of Sydney and Sydney Local Health District, Sydney, NSW, Australia; ^5^ Department of Cardiology, St Vincent's Hospital, Sydney, NSW, Australia; ^6^ Department of Rheumatology, St Vincent's Hospital, Sydney, NSW, Australia; ^7^ St Vincent’s Centre for Applied Medical Research, Sydney, NSW, Australia

**Keywords:** gout, hyperuricaemia, heart transplant (HTx), lung transplant (LTx), heart/lung transplant, uric acid

## Abstract

**Introduction:**

Gout may complicate solid organ transplantation with potentially serious consequences. An accurate prevalence of gout in this population is unknown.

**Objectives:**

This study aimed to estimate the prevalence of gout in the heart and/or lung transplantation population through a systematic review and meta-analysis.

**Methods:**

MEDLINE, Embase, PsycINFO, CENTRAL and Cochrane Library (inception to February 2022) were searched for studies that reported the prevalence and/or incidence of gout in heart and/or lung transplant recipients. Two authors extracted outcomes data. Data were pooled using a random effects model. Overall quality of evidence was assessed using GRADE. Primary outcomes were the prevalence of pre- or post-transplant gout expressed as a prevalence rate (95% CI). Secondary outcomes included risk factors for gout, adverse events, and therapeutic complications of gout treatment.

**Results:**

Ten studies were included. Gout prevalence (PR) was 8% pre-transplant (PR = 0.08; 95% CI: 0.05–0.12; 4 studies *n* = 651) and 6% post-transplant (PR = 0.06; 95% CI: 0.06–0.06; 10 studies *n* = 45,298). Post-transplant gout prevalence in heart transplant recipients was almost three times higher than lung transplant recipients (PR = 0.16; 95% CI: 0.13–0.20 vs. PR = 0.06; 95% CI: 0.05–0.06 respectively). Patients with a pre-transplant history of gout had a higher risk of developing post-transplant gout than patients without (RR = 3.61; 95% CI: 2.19–5.95). Factors associated with gout and outcomes for heart and/or lung transplant recipients with gout were comprehensively reviewed from the included studies.

**Conclusion:**

Gout is highly prevalent in heart and/or lung transplant patients. Pre-transplant gout is predictive of developing symptomatic post-transplant gout. This has significant implications for management of heart/lung transplant patients.

**Systematic Review Registration:**

https://www.crd.york.ac.uk/, PROSPERO (CRD42020190632).

## Introduction

Gout is an inflammatory arthritis caused by tissue deposition of monosodium urate. While this is a chronic process, gout may cause acute attacks, characterised by sudden onset pain, tenderness, swelling, erythema, and warmth of affected joints/tissues. Gout is a significant health issue due to its association with cardiovascular, renal and metabolic disease, and overall reduced life expectancy (1, 2). Gout carries a substantial global burden of disease with an estimated worldwide prevalence of up to 4% of the global population (3) and has progressively increased over time in some countries (4).

Solid organ transplant recipients have an increased risk of developing gout, which may be explained by both the underlying pathology and medications used to treat these conditions. For example, heart transplant recipients may have concurrent renal impairment, or develop hypoxia-induced uric acid synthesis (5–7), increasing their susceptibility to hyperuricaemia. Additionally, administering loop and thiazide diuretics to heart failure patients can decrease renal uric acid excretion, resulting in hyperuricemia and an increased susceptibility to pre-transplant gout (5–8). Immunosuppressive medications may drive hyperuricaemia themselves, or have significant interactions with pharmaceuticals used to treat acute gout or lower serum urate (9–16).

Given the risk factors outlined above, gout remains an important, yet underappreciated cause of morbidity in the heart/lung transplantation population. An accurate estimate of the burden of gout in this population is required to address this significant issue.

## Objective

Although the current literature acknowledges the presence of gout in heart/lung transplant recipients, there remains a paucity of studies assessing the prevalence of gout in heart or lung transplant patients. Furthermore, risk factors and outcomes for gout in the heart/lung transplant cohort have not been assessed in depth. To date, this is the first systematic review and meta-analysis that has attempted to quantitate gout prevalence in this population.

## Methods

The protocol was registered on PROSPERO on 5 June 2020 (receipt number: 190632). This review was conducted in accordance with PRISMA guidelines (17).

## Design

### Types of studies

Observational studies (e.g., cohort studies, case-control studies, cross-sectional studies) reporting on the incidence or prevalence of gout in individuals who had undergone heart, lung or heart and lung transplantation were included. There were no restrictions for language and translations were attempted for non-English published articles/data. As clinical diagnosis of gout has remained largely unchanged for many decades, there was no restriction on the year of publication.

### Participants

Studies were eligible if they included patients who had undergone a heart transplant, lung transplant, or heart-lung transplant. Studies were excluded if they did not explicitly mention gout as a comorbidity, adverse event or an outcome.

### Comparison

The “gout” group consisted of heart and/or lung transplant patients who had a gout flare before and/or after their transplant. The “no gout” group consisted of heart and/or lung transplant patients who never had a gout flare before or after transplantation.

### Electronic searches

The search strategy was developed by [redacted] and edited by [redacted]. A search was performed in MEDLINE, Embase, PsycINFO, CENTRAL and Cochrane Library (all from inception to February 2022, without language restrictions) for eligible reports. Reference lists of relevant observational studies were screened. Search terms included “gout” AND “transplantation” OR “heart transplant” OR “lung transplant”.

### Study selection

Four independent reviewers [redacted] screened titles and abstracts. Six reviewers [redacted] independently inspected the full manuscript of potentially eligible observational studies to determine eligibility.

### Assessment of heterogeneity

Clinical heterogeneity was assessed by comparing participant characteristics, type and dosage of immunosuppressive medications, duration of follow-up, method of gout diagnosis, and the type and dosage of gout medications.

### Overall quality of evidence rating

The Gradings of Recommendations Assessment, Development and Evaluation (GRADE) method was used for evaluating overall quality of evidence (18). Baseline quality of evidence was reported as “high” and downgraded a level for each of the four factors: limitations in study design, result inconsistency [wide variance of point estimates across studies or if statistical heterogeneity between trials was large (*I*^2 ^> 50)] (19), result imprecision (wide confidence intervals, total sample size less than <300), and publication bias (assessed using funnel plot analysis/Egger's regression test for 10 or more studies). It was not necessary to downgrade for indirectness as this review encompassed a specific review question. Overall quality of evidence was rated as “high”, “moderate”, “low” or “very low”.

## Data collection

Two reviewers (BC, CAS) extracted data using piloted extraction forms. Other investigators were also consulted (RD, LG, RP, EU). Non-English articles were translated. Information on outcomes data and study characteristics were collected.

## Bias assessment

Two reviewers (BC, CAS) independently assessed the risk of bias. Cohort studies were assessed using the Newcastle-Ottawa Scale (NOS) for quality assessment of non-randomised studies (20). Studies with a score of >7 or higher were deemed to have a low risk of bias, studies with a score ≤6 were deemed to have a high risk of bias. Cross-sectional studies were assessed using an adapted version of Hoy et al.'s risk of bias tool for prevalence studies (21). Studies were classified as having low, moderate, or high risk of bias.

## Outcome measures

The primary outcome was to assess gout prevalence in people undergoing heart and/or lung transplantation, pre- and post-transplant. These include gout flares, intercritical gout (i.e., between flares) and chronic gouty arthritis as defined by the European League Against Rheumatism (EULAR) (22).

The secondary outcomes were risk factors, adverse events, therapeutic complications and transplant-related mortality in heart and/or lung transplant recipients with gout.

Adverse events data included: interval between transplant and gout flare, sites of gout flare and tophi formation, duration of gout, complications of gout, infection and acute rejection episodes. Serum urate levels and renal function in the post- heart and/or lung transplant gout cohort were collected.

### Subgroup analysis

A sub-group analysis compared gout prevalence in heart transplant patients with lung transplant patients. Patients with no history of pre-transplant gout were compared with patients with a history of pre-transplant gout. The pre-transplant prevalence of gout was compared with post-transplant prevalence of gout.

## Data synthesis

The meta-analysis and subgroup analysis was carried out using Comprehensive Meta-Analysis random-effects model Version 3 (23). Prevalence ratios (PR) were expressed as the total number of transplant patients with gout over the total number of transplant patients. Results for dichotomous data were presented as risk ratio (RR) with 95% confidence intervals (CI). Results not able to be pooled are described descriptively.

## Results

### Study selection

**Figure 1:**
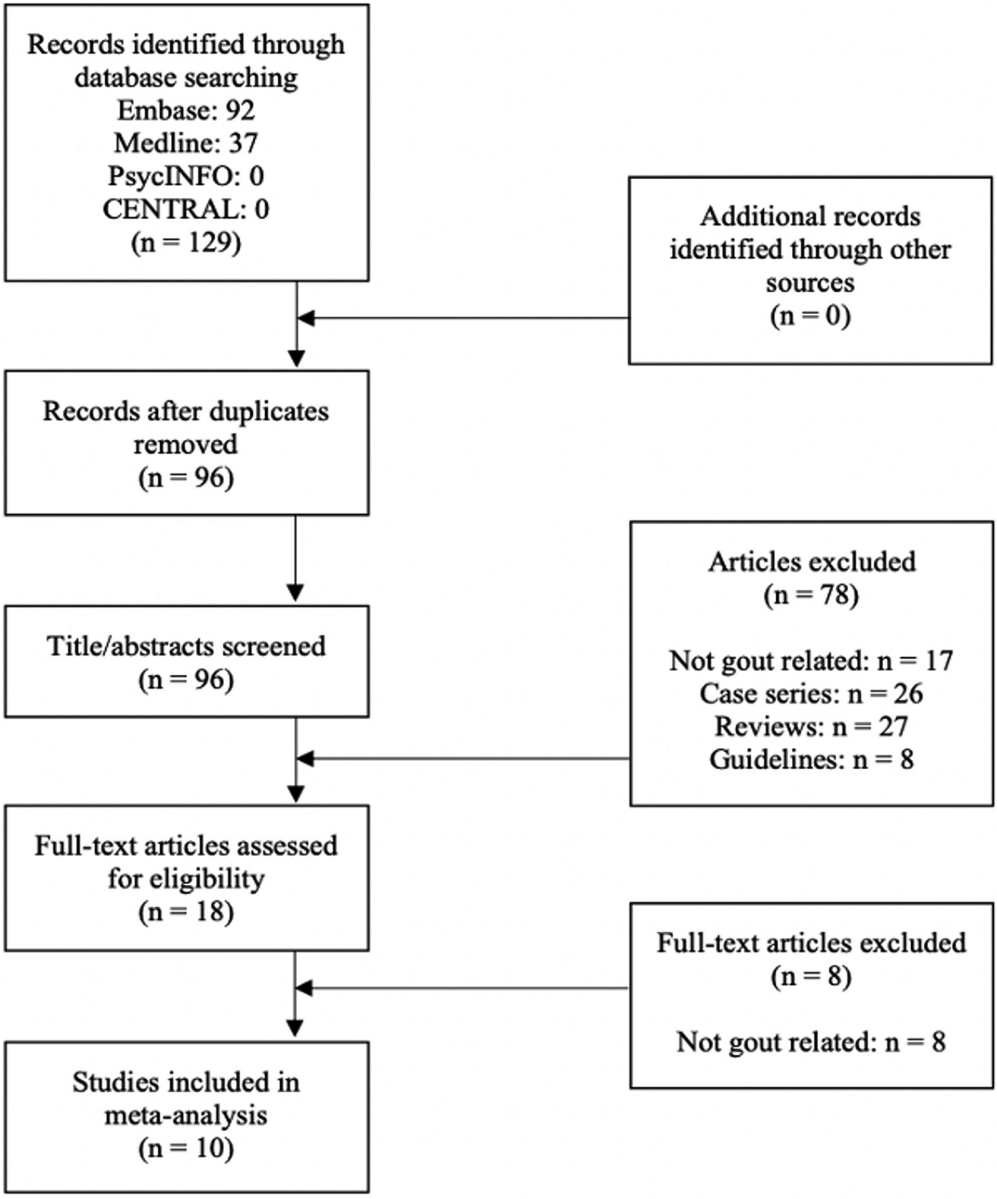
Study flow diagram according to PRISMA guidelines.

A total of 129 studies were identified from the searches. After duplicate articles were removed, 96 articles were included in title/abstract screening. Eighteen articles were deemed relevant for a full-text review, of which 10 articles met eligibility criteria (24–33). Reasons for exclusion of the eight studies after full-text review are described in Figure 1.

There were data of sufficient quality to perform a metaanalysis on gout prevalence and the association of premorbid gout with post-transplant gout flare (GRADE ratings in Supplementary Tables S3–S5).

### Characteristics of included studies

Included studies are summarised in Table 1. Seven were retrospective cohort studies (24–27, 29–31), one was a prospective cohort study (28), and two were cross-sectional studies (32, 33).

**Table 1:** Characteristics of included studies.

Study	Setting	Transplant type	Study aims	Study type	Selection criteria	Sample size	Mean age	Gender	Immunosuppressive regimen	Mean follow-up duration (cohort)/ time since transplant (cross-sectional)	Industry involvement	Gout Assessment method
Aravot et al. (24)	Harefield Hospital, London, England	Heart	General post- transplant symptoms	Retrospective cohort	Heart transplant patients aged between 60 and 69, under a cyclosporine-azathioprine regimen between June 1983 and January 1988	25 (heart)	63 years (range: 60–69)	24 male, 1 Female	Pre-operative: Cyclosporine (2–10 mg/kg), azathioprine (2 mg/kg), Intraoperative: 1g of methylprednisolone Post-operative: cyclosporine b.d. (Varied between 2 and 20 mg/kg/day), azathioprine (2 mg/kg/day)	22 months (range: 5–59 months)	NR	NR
Brigham et al. (25)	United States (outpatients)	Heart, lung, kidney, liver	Gout prevalence	Retrospective cohort	Estimated surviving population of transplant recipients who received a transplant between 1988 and 2017. Annual cohort sizes were combined with published survival rates for each cohort.	29,300 (heart) 14,700 (lung)	NR	NR	NR	Range: 0–29 years	Yes. Funded by Horizon Pharma	Insurance claims Data
Burack et al. (26)	University of Pittsburgh, Pittsburgh, USA	Heart, heart-lung	Gout prevalence	Retrospesctive cohort	All surviving patients who received a heart/heart-lung transplant between June 1980 and December 1986. Patients receiving colchicine prophylaxis were excluded.	189 (heart or heart- lung)	NR	NR	Cyclosporine (99%–100%), azathioprine (71%–83%), prednisolone (100%). Dosage was not reported.	No pre-transplant gout: range from 2.3–3.7 years Had pre-transplant gout: mean surveillance period of 27.6 months	NR	Self-reported
Farge et al. (27)	Hôpital Broussais & Hôpital Lariboisière, Paris, France	Heart	Gout prevalence	Retrospective cohort	NR	117 (Heart)	NR	NR	Triple therapy, low- dosage regimen consisting of steroids, azathioprine and cyclosporine. Cyclosporine was only introduced 3 days post-transplant.	12.8 ± 8.5 months	NR	NR
Grady et al. (28)	United States (outpatient, multiple sites)	Heart	General post- transplant symptoms	Prospective cohort	Participants from a longitudinal study of quality of life outcomes.	208 (Heart)	53.8 ± 9.9 years	78% male 22% female	Triple therapy consisting of cyclosporine (84%) or tacrolimus (16%), mycophenolate mofetil (52%) or azathioprine (48%), and steroids. Dosage was not specified	10 years	No	Self-reported
Manche (29)	Mater Dei University Hospital, Malta	Heart	General post- transplant symptoms	Retrospective cohort	All transplants performed in Malta since 1995 (including 1 jointly performed in London). Intra- operative deaths were excluded.	13 (heart)	55.3 years (range: 15–63)	NR	Triple therapy consisting of methylprednisolone IV (changed to PO prednisolone after a few days), azathioprine and cyclosporine. Dosage was not specified.	Range: 1–15 years	NR	NR
Rozenberg et al. (32)	Hôpital de la Pitié- Salpétrière, Paris, France	Heart	Post-transplant rheumatological symptoms	Cross- sectional	Consecutive heart transplant recipients.	365 (heart)	45.9 ± 12.0 years (range: 11–68)	292 male 73 female	Triple therapy consisting of cyclosporine, azathioprine and prednisolone. Dosage was not specified.	35.8 ± 25.6 months (range: 1–115)	NR	Physician diagnosis
Shibolet et al. (30)	Hadassah University Hospital, Jerusalem, Israel	Heart, liver	Gout prevalence	Retrospective cohort	Consecutive transplant recipients in a single transplantation centre, with at least 3 years of follow-up.	47 (heart)	57 years (range: 23–78)	41 male 6 female	Cyclosporine (95.9%), tacrolimus (4.1%), steroids (100%), azathioprine (66%), mycophenolate mofetil (4.3%). Dosage was not specified.	105.6 months (range: 37–225)	NR	Medical records
Wagener et al. (33)	Hannover Medical School, Hanover, Germany	Heart	Post-transplant rheumatological symptom	Cross- sectional	Patients who had a rheumatological examination performed during follow-up.	120 (heart)	Without rheumatological symptoms: 41 years. With rheumatological symptoms: 47 years.	105 male 15 female	Cyclosporine, azathioprine, prednisolone. Dosage was not specified.	Without rheumatological symptoms: 25 months With rheumatological symptoms: 26 months	NR	Physician diagnosis
Wluka et al. (31)	Alfred Hospital, Melbourne, Australia	Heart	Gout prevalence	Retrospective cohort	Patients who had undergone orthotopic heart transplantation before July 31, 1998, and lived in Victoria, Australia.	225 (heart)	49.4 years (range: 15.8–66.1)	183 male 42 female	Cyclosporine, azathioprine, prednisolone. Dosage was not specified.	50.8 months (range: 0–111.2 months; SD: 36)	NR	Medical records

Among the 5/10 studies that characterised age and/or gender, mean age ranged from 41 to 63 years and most patients were male. The follow-up period ranged from 1 to 15 years. Nine studies provided details of immunosuppressants administered to transplant recipients (24, 26–33) 7/10 studies explicitly reported their methods to identify gout (Table 1) (25, 26, 28, 30–33).

### Risk of bias

All eight cohort studies were assessed to have a high risk of bias (Supplementary Table S1) (24–31). Both cross-sectional studies were assessed to have a low risk of bias (Supplementary Table S2) (32, 33).

### Prevalence of pre-transplant gout in heart and/or lung transplant patients

**Figure 2:**
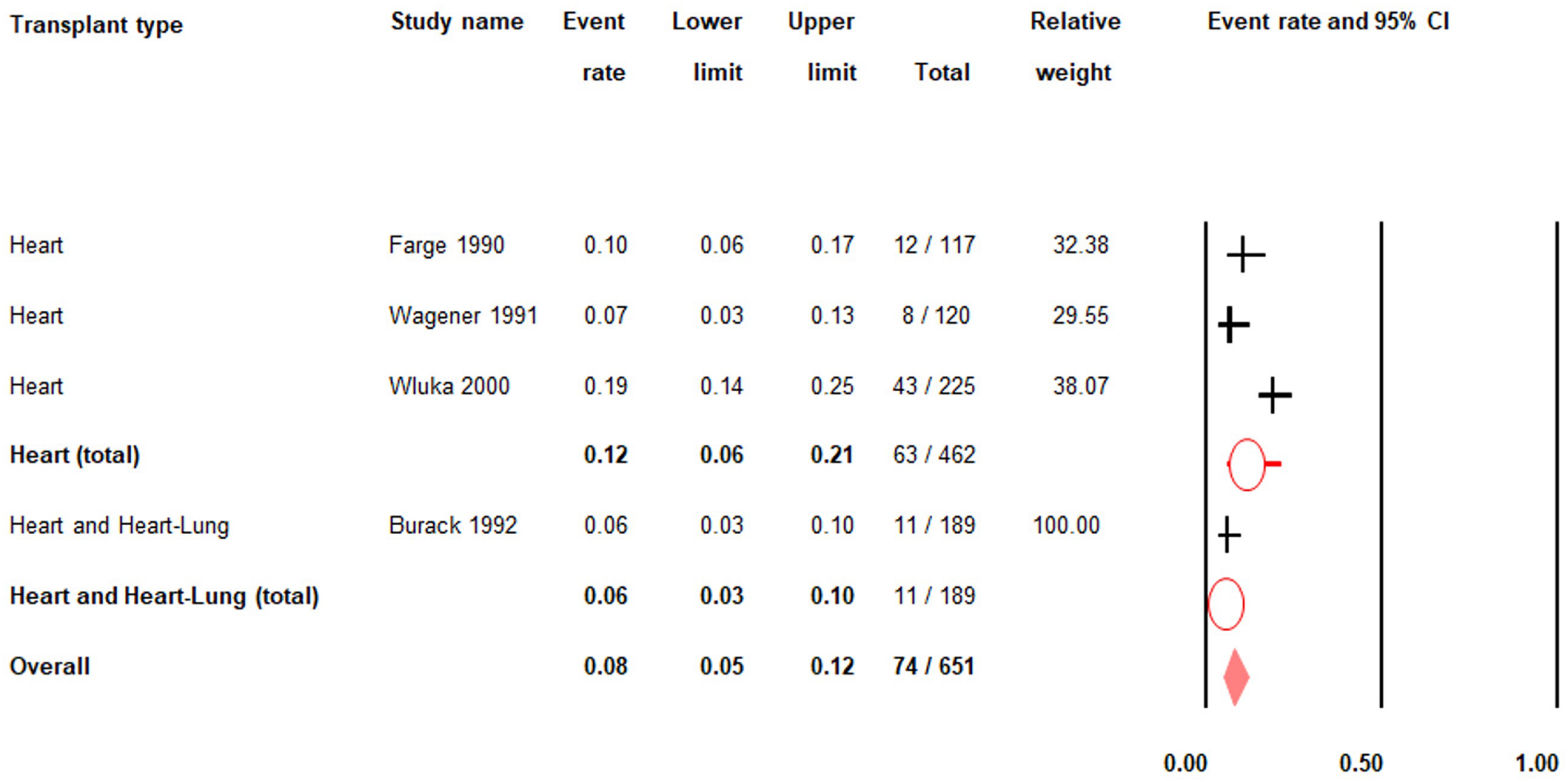
Pre-transplant prevalence of gout. 95% CI; |: prevalence rate; O: total prevalence rate for each transplant type; ♦: overall prevalence rate.

There was low quality evidence from four studies (*n* = 651) that the pre-transplant prevalence of gout in heart and/or heart-lung transplant patients was 8% (PR = 0.08; 95% CI: 0.05–0.12) (Figure 2; Supplementary Table S3) (26–28, 31–33).

Among heart transplant patients only, there was very low quality evidence from three studies (*n* = 462) that the pre-transplant prevalence of gout was 12% (PR = 0.12; 95% CI: 0.06–0.21) (Figure 2; Supplementary Table S3) (27, 28, 31, 33).

There was moderate quality evidence from one study (*n* = 189) that the pre-transplant prevalence of gout in heart and heart-lung transplant patients was 6% (PR = 0.06; 95% CI: 0.03–0.10) (Figure 2; Supplementary Table S3) (26). There were no data on the pre-transplant prevalence of gout in patients who had lung transplants only.

### Prevalence of post-transplant gout in heart and/or lung transplant patients

**Figure 3:**
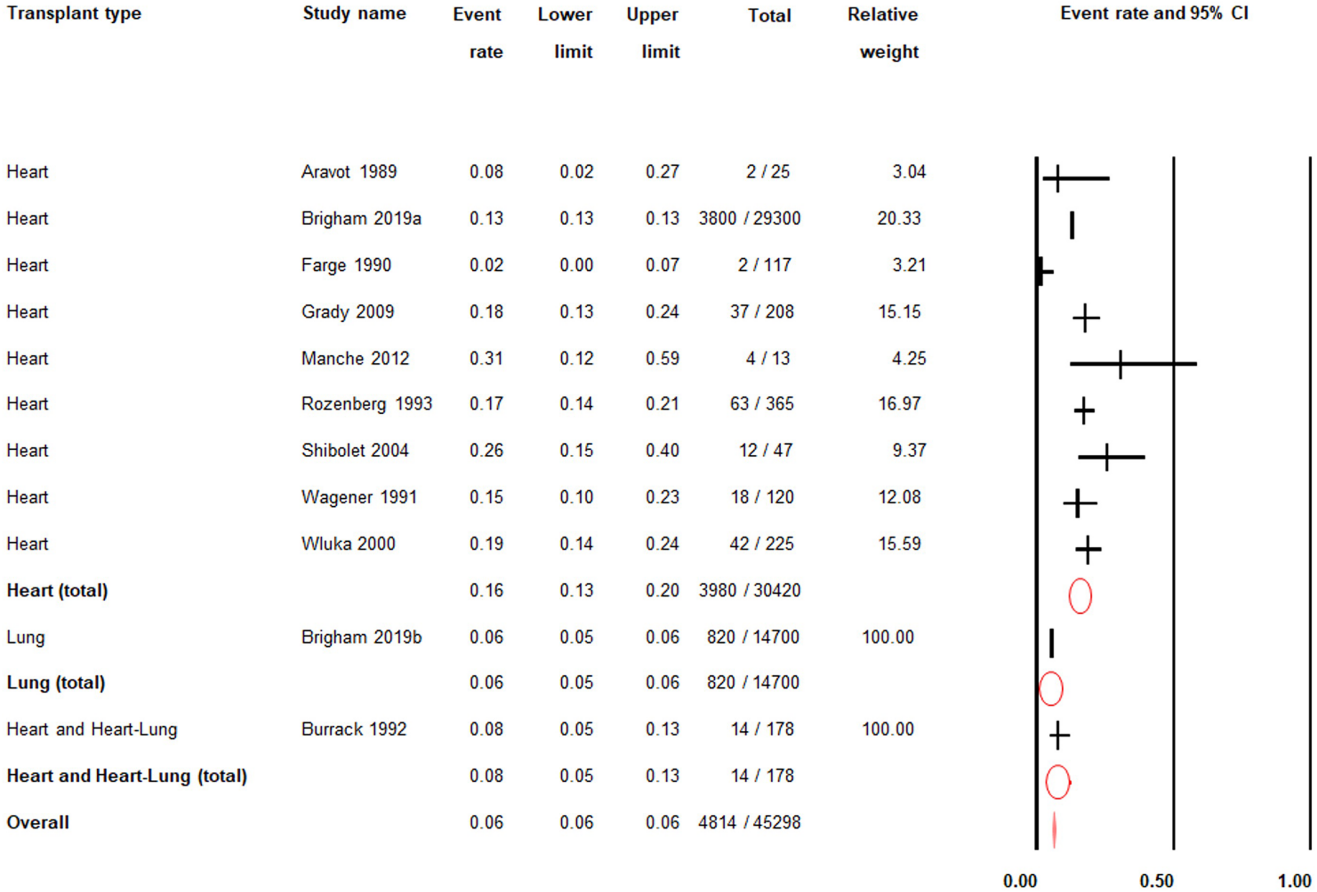
Post-transplant prevalence of gout. 95% CI; |: prevalence rate; O: total prevalence rate for each transplant type; ♦: overall prevalence rate.

There was low quality evidence from ten studies (*n* = 45,298) that the post-transplant prevalence of gout was 6% (PR = 0.06; 95% CI: 0.06–0.06) (Figure 3; Supplementary Table S4) (24–33).

Among heart transplant patients only, there was low quality evidence from nine studies (*n* = 30,420) that the post-transplant prevalence of gout was 16% (PR = 0.16; 95% CI: 0.13–0.20) (Figure 3; Supplementary Table S4).

Among lung transplant patients only, there was moderate quality evidence from one study (*n* = 14,700) that the post-transplant prevalence of gout was 6% (PR = 0.06; 95% CI: 0.05–0.06) (Figure 3; Supplementary Table S4).

Among heart and heart-lung transplant patients, there was moderate quality evidence from one study (*n* = 178) that the post-transplant prevalence of gout was 8% (PR = 0.08; 95% CI: 0.05–0.13) (Figure 3; Supplementary Table S4).

### Relative risk of post-transplant gout

There was moderate quality evidence from two studies (*n* = 342) that the relative risk of experiencing a post-transplant gout flare was higher in patients who had a pre-transplant history of gout than patients who had no prior history of gout (RR = 3.61; 95% CI: 2.19–5.95) (Supplementary Figure S1; Supplementary Table S5) (27, 31).

### Risk factors for gout development in transplant recipients

4/10 studies reported the mean ages of gout patients (26, 29, 31, 32). Two of these studies reported that patients with gout were significantly older than patients without gout, and a significantly higher prevalence of gout was seen in males compared to females (31, 32).

4/10 studies reported that diuretics were used more commonly among heart transplant recipients with gout compared to heart transplant recipients without gout (26, 30–32). One study reported statistically significant findings (31).

### Characteristics of post-transplant populations with gout

#### Interval between operation and gout flare

3/10 studies reported the duration between transplant and gout flare (26, 27, 31). One study reported a mean of 17 months between transplantation and gout flare (range: 1–41) (26). Another study reported an interval of 6 months between operation and gout flare in recipients with pre-transplant gout, and 18 months in recipients with new-onset gout (27). A third study reported a mean of 25.9 months in patients with pre-transplant gout and 43.9 months in patients with new-onset gout (31).

None of the studies mentioned if any patient suffered from an inpatient gout flare, i.e., immediately post-transplant before their initial discharge from hospital.

#### Site of gout flares and tophi formation

2/10 studies reported the site of gout flares in post-transplant populations (26, 27). In both studies, the first metatarsophalangeal joint was most commonly affected. Other joints affected included the midtarsal, ankle, elbow, wrist, and small hand joints (26).

Tophi formation was reported in 4/10 studies (26, 27, 31, 32). In one study, tophi formation was seen in 6/14 (42.9%) of new-onset gout patients and 2/11 (18.2%) of those with recurrent gout. In three other studies, tophaceous gout was seen 7.9%–50% of patients (27, 31, 32).

#### Symptom duration

No study reported the frequency or duration of episodes of flare.

#### Articular complications

2/10 studies reported articular complications in post-transplant populations (26, 32). Bacterial infections of the joint, bursa, or tophi occurred after transplantation in 1/20 (5%) of recipients with recurrent gout, and 3/20 (15%) of recipients with new-onset gout (26). One patient required a surgical debridement because of a bacterial superinfection of a tophus in the olecranon bursa. 2/63 (3.2%) of patients with gout showed signs of osteoarticular damage (32).

#### Mortality

1/10 studies compared mortality rates between patients with gout and patients without gout (31). The mortality rate was 4/23 (17.4%) in patients with new-onset gout, 3/19 (15.8%) in patients with recurrent gout, and 45/159 (28.3%) in patients who never had gout.

#### Therapeutic complications of gout medications

3/10 studies reported changes to immunosuppressants directly because of gout medications (27, 29, 31). One study stated that azathioprine was progressively discontinued before allopurinol could be initiated (27). In a second study, azathioprine was switched to mycophenolate mofetil when allopurinol was introduced in a patient with gout (29). In another study, azathioprine was ceased with cyclophosphamide or mycophenolate substituted in 5/6 (83%) of those with pancytopenia, and in 9/18 (50%) without pancytopenia (31).

#### Hyperuricaemia

3/10 studies reported the mean serum urate levels in heart transplant recipients (26, 30, 32). The prevalence of hyperuricaemia among heart transplant recipients ranged from 72%–100%. Serum urate was reported to increase post-transplant; other factors associated with an elevated serum urate were cyclosporine use, diuretic use, and tophaceous gout (26, 32).

## Discussion

While gout has been a recognised comorbidity in heart and/or lung transplantation for decades (34), this review is the first to characterise gout prevalence in heart and/or lung transplant patients in the literature.

The results of this study highlight the significant risk of gout in heart and lung transplant populations. This study reported low-quality evidence that the pre- and post-transplant prevalence of gout in heart and/or lung transplant patients was 8% and 6% respectively. In comparison, the estimated all-age prevalence of gout in western countries is between 0.5% and 5% of the general population (35). The increased prevalence of gout in the heart and lung transplant cohort likely reflects the pre-transplant disease state and medication use (e.g., cardiac failure and subsequent diuretic use) which increases susceptibility to hyperuricemia.

This study reported medium quality of evidence that the risk of post-transplant gout is greater in patients with pre-transplant gout compared to patients without pre-transplant gout. This augments the previous observation that flares of gout occur earlier post-transplant in patients with pre-existing gout (27, 31).

The risk factors for gout development in post-transplant populations are similar to the general population. Multiple studies reported that patients with gout were significantly older than patients without gout, and males were more likely to develop gout compared to females (27). Diuretics remain a key risk factor, and were used more commonly in heart transplant recipients with gout compared to recipients without gout (31, 32).

Treatment of gout may itself give rise to additional complications in this population. Calcineurin inhibitors (CNI) may contribute to hyperuricaemia and pose significant drug-drug interactions with agents used to treat gout flares, such as colchicine. The potentially serious interaction of azathioprine with xanthine oxidase inhibitors (e.g., allopurinol) is well-described. However, modern immunosuppressants may overcome some of these issues: for example, mycophenolate mofetil, which does not interact with allopurinol, is increasingly used in place of azathioprine. Nonetheless, azathioprine may still be used in certain clinical scenarios and as such clinicians must be aware of this significant interaction.

This study had several strengths. Firstly, it is the first meta-analysis to assess gout prevalence in the heart/lung transplant population. The study population was extracted from heterogenous clinical settings, and the characteristics of post-transplant recipients with gout were assessed in detail.

Limitations to this study include the large statistical heterogeneity between studies, which resulted in considerable variance in gout prevalence. While this study did not place limits on study age, the clinical diagnosis of gout has remained essentially unchanged over the study period and thus study age has limited impact on estimation of gout prevalence in this regard. Only one study mentioned gout prevalence in lung transplant recipients (26, 30–32), hindering a direct comparison between heart and lung transplant patients. There are limited data on the prevalence of pre-transplant gout: one study specifically excluded patients with pre-transplant gout (25), while another study excluded patients with pre- transplant gout when characterising patients with post-transplant gout (30). The true prevalence of gout may be confounded by the lack of standardisation in the diagnosis of gout. Finally, most of the selected studies did not perform multivariate analyses to assess the significance of potential risk factors such as age, race, gender, or comorbidities such as renal impairment.

To improve the quality of evidence of gout prevalence in these populations, future heart/lung transplantation studies would benefit from use of established gout diagnostic criteria (36), using a sufficient duration of follow up to capture incident gout (31), report gout incidence over regular time periods (e.g., monthly intervals post-transplantation) and capture gout attacks in the immediate post-transplantation period. Serum urate should be determined regularly pre-and post-transplant. Future studies should include gout as an outcome measure to allow tracking of gout prevalence over time, particularly as immunosuppressive treatment and other factors that influence hyperuricaemia change. For example, the prevalence of hyperuricaemia and gout may increase as transplant candidacy guidelines permit patients with renal dysfunction; furthermore, the background burden of gout appears to be increasing which may be mirrored in transplant populations (4, 37). There is also a paucity of data of the prevalence of gout in the combined heart-kidney, lung-kidney and thoracoabdominal triple organ transplant setting.

The prevalence of gout in heart/lung transplant populations as determined in this study is higher than that reported in the general population. In context of the increasing worldwide prevalence of gout, guidelines for managing gout in this population are paramount for the guidance of future practice. However, despite major advancements in gout therapy and guidelines published by the American College of Rheumatology (ACR) (38) to inform gout management, there are no specific guidelines on the management of gout in the setting of concurrent immunosuppressive therapy in heart and/or lung transplant patients. Notably, the International Society for Heart and Lung Transplantation (ISHLT) Guidelines for the Care of Heart Transplant Recipients recommends the use of anti-hyperuricaemic therapies for gout in heart transplant patients; the results of this study provide an accurate estimate of gout burden in this population to support this recommendation (39). Finally, awareness of gout prevalence and the potential pitfalls in gout management in this population would serve to improve patient outcomes and safety. Adoption of an anticipatory approach, or screening transplant patients for underlying hyperuricaemia or gout, may improve patient outcomes and would benefit from further study.

There is considerable pre- and post-transplant prevalence of gout in heart and lung transplantation recipients. Pre-existing gout increases the risk of a post-transplant gout flare. Addressing the factors that drive prevalence, as well as the management of gout, are significant areas of unmet need in the heart/lung transplantation population.

## Data Availability

The original contributions presented in the study are included in the article/**Supplementary Material**, further inquiries can be directed to the corresponding author.
