# Supplementary material for: Meta-analysis and systematic review of gout prevalence in the heart/lung transplantation population
Source: Front Transplant. 2024 May 20;3:1356058. doi: 10.3389/frtra.2024.1356058 (PMC11235269; doi:10.3389/frtra.2024.1356058)
Supplement: Supplementary file 1 [file Datasheet1.docx]

**Supplementary Appendix Material**

**Supplementary Table 1: Newcastle-Ottawa Scale risk of bias assessment for cohort studies.**

|  | **SELECTION** | | | | **COMPARABILITY** | **OUTCOME** | | | |
| --- | --- | --- | --- | --- | --- | --- | --- | --- | --- |
| **Study** | **Representativeness of the exposed cohort** | **Selection of the non-exposed cohort** | **Ascertainment of exposure** | **Demonstration that outcome of interest was not present at start of study** | **Comparability of cohorts on the basis of the design or analysis** | **Assessment of outcome** | **Was follow-up long enough for outcomes to occur** | **Adequacy of follow up of cohorts** | **Overall rating (out of 9)*** |
| Aravot 1989 | 1 | N/A | 1 | 0 | 0 | 0 | 1 | 0 | **3** |
| Brigham 2019 | 1 | N/A | 1 | 0 | 0 | 1 | 1 | 0 | **4** |
| Burack 1992 | 1 | N/A | 1 | 0 | 0 | 0 | 1 | 0 | **3** |
| Farge 1990 | 0 | N/A | 1 | 0 | 0 | 0 | 1 | 0 | **2** |
| Grady 2009 | 1 | N/A | 1 | 0 | 0 | 0 | 1 | 1 | **4** |
| Manche 2012 | 1 | N/A | 1 | 0 | 0 | 0 | 1 | 0 | **3** |
| Shibolet 2004 | 1 | N/A | 1 | 1 | 0 | 1 | 1 | 1 | **6** |
| Wluka 2000 | 1 | N/A | 1 | 0 | 0 | 1 | 1 | 1 | **5** |

Legend: *: Overall rating

- ≥ 7: high quality
- ≤ 6: low quality

**Supplementary Table 2: Hoy et al. risk of bias assessment for cross-sectional studies.**

|  | **EXTERNAL VALIDITY** | | | | **INTERNAL VALIDITY** | | | | | | |
| --- | --- | --- | --- | --- | --- | --- | --- | --- | --- | --- | --- |
| **Study** | **Representative of study population** | **Sampling frame representative** | **Random selection / undertaken census** | **Minimum non-response bias** | **Data collected directly from subjects** | **Acceptable case definition** | **Reliable and valid study instrument** | **Same mode of data collection for all subjects** | **Appropriate length of shortest prevalence period** | **Appropriate numerator and denominator for parameter of interest** | **Summary risk of bias*** |
| Rozenberg 1993 | Yes | Yes | No | Yes | Yes | Yes | Yes | Yes | No | Yes | **Low risk** |
| Wagener 1991 | Yes | Yes | No | Yes | Yes | Yes | Yes | Yes | No | Yes | **Low risk** |

Legend: *: Summary risk of bias

- Low risk of bias: Further research is very unlikely to change the confidence in the estimate.
- Moderate risk of bias: Further research is likely to have an important impact on the confidence in the estimate and may change the estimate.
- High risk of bias: Further research is very likely to have an important impact on the confidence in the estimate and is likely to change the estimate.

**Supplementary Table 3: Summary of effect size estimates and GRADE ratings for pre-transplant gout.**

| **Number of studies** | **Total sample size** | **Effect estimate; PR (95% CI)** | **GRADE** | | | | |
| --- | --- | --- | --- | --- | --- | --- | --- |
|  |  |  | **Study limitation** | **Imprecision** | **Inconsistency (I^2^)** | **Publication bias** | **GRADE** |
| Pre-transplant (heart transplant only) | | | | | | | |
| 3 | 462 | 0.12 (0.06-0.21) | Yes | Yes | Yes (82.0%) | N/A | **Very low** |
| Pre-transplant (heart +/- lung transplant) | | | | | | | |
| 1 | 189 | 0.06 (0.03-0.10) | Yes | No | No (0%) | NA | **Moderate** |
| Pre-transplant (overall) | | | | | | | |
| 4 | 651 | 0.08 (0.05-0.12) | Yes | No | Yes (85.0%) | N/A | **Low** |

Legend: PR: prevalence rate, CI: confidence interval, N/A: not applicable

**Supplementary Table 4: Summary of effect size estimates and GRADE ratings for post-transplant gout.**

| **Number of studies** | **Total sample size** | **Effect estimate; PR (95% CI)** | **GRADE** | | | | |
| --- | --- | --- | --- | --- | --- | --- | --- |
|  |  |  | **Study limitation** | **Imprecision** | **Inconsistency (I^2^)** | **Publication bias** | **GRADE** |
| Post-transplant (heart transplant only) | | | | | | | |
| 9 | 30420 | 0.16 (0.12-0.20) | Yes | No | Yes (77.1%) | N/A | **Low** |
| Post-transplant (lung transplant only) | | | | | | | |
| 1 | 14700 | 0.06 (0.05-0.06) | Yes | No | No (0%) | N/A | **Moderate** |
| Post-transplant (heart +/- lung transplant) | | | | | | | |
| 1 | 178 | 0.08 (0.05-0.13) | Yes | No | No (0%) | N/A | **Moderate** |
| Post-transplant (overall) | | | | | | | |
| 10 studies  (11 comparisons) | 45298 | 0.06 (0.06-0.06)* | Yes | No | Yes (98.3%) | No (Egger p-value = 0.95) | **Low** |

Legend: PR: prevalence rate, CI: confidence interval, N/A: not applicable, *: All results were rounded up to 2 decimal places for consistency. Actual post-transplant PR (overall) = 0.060 (95% CI: 0.056-0.064).

**Supplementary Table 5: Summary of effect size estimates and GRADE ratings for post-transplant gout in patients with/without pre-transplant gout.**

| **No. studies** | **Total sample size** | **Effect estimate; RR (95% CI)** | **GRADE** | | | | |
| --- | --- | --- | --- | --- | --- | --- | --- |
|  |  |  | **Study limitation** | **Imprecision** | **Inconsistency (I^2^)** | **Publication Bias** | **GRADE** |
| Post-transplant (heart transplant only) | | | | | | | |
| 2 | 342 | 3.61 (2.19-5.95) | Yes | No | No (0%) | N/A | **Moderate** |

Legend: RR: relative risk, CI: confidence interval, N/A: not applicable

**Supplementary Figure 1: Relative risk of developing post-transplant gout.**


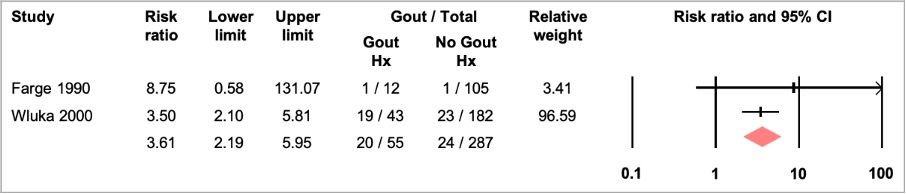
Legend: — : 95% CI; **|** : prevalence rate; ♦ : overall prevalence rate
